# Finite Element Modeling of Quenching Residual Stress in GH4169 Alloy Turbine Disk Forgings

**DOI:** 10.3390/ma19214544

**Published:** 2026-10-28

**Authors:** Haoyu Lu, Shengjie Wang, Yang Li, Haijiang Dong, Ying Yu, Shilei Li, Chunlei Zhao

**Affiliations:** 1 China United Test & Certification Co., Ltd., Beijing 101407, China; luhaoyu@cutc.net (H.L.); liyang@gbtcgroup.com (Y.L.); donghaijiang@cutc.net (H.D.); yuying@cutc.net (Y.Y.); lishilei@ustb.edu.cn (S.L.); 2 United (Chongqing) Test & Certification Co., Ltd., Chongqing 400700, China; 3 Guobiao (Beijing) Testing & Certification Co., Ltd., Beijing 101407, China; 4 State Key Laboratory for Advanced Metals and Materials, University of Science and Technology Beijing, Beijing 100083, China

**Keywords:** residual stress, finite element simulation, GH4169 superalloy, turbine disk

## Abstract

A finite element model was established to investigate the formation mechanism and evolution behavior of quenching residual stress in GH4169 alloy turbine disk forgings, and the model was validated by X-ray diffraction and hole-drilling measurements. The results show that the evolution of quenching residual stress can be divided into three stages, and the stress distribution is closely related to the temperature gradient and plastic deformation. During quenching, the stress state gradually changes from “inner compression–outer tension” to “inner tension–outer compression”. After quenching, the residual stress field is characterized by surface compressive stress and internal tensile stress, with the maximum compressive and tensile stresses reaching about −794 MPa and +720 MPa, respectively. Both equivalent stress and equivalent plastic strain show high values in the surface and core regions and low values in the transition region. Their maximum values appear at the disk surface, reaching 733.4 MPa and 6.018%, respectively. Further analysis indicates that the spatial and temporal evolution of plastic strain is strongly dependent on the temperature gradient. The incompatible plastic deformation induced by the temperature gradient is the dominant mechanism for quenching residual stress formation, while the accumulation of plastic strain directly determines the magnitude and distribution of the residual stress. The relative deviation between the simulated and measured residual stresses is about 15%, which is within the acceptable range for engineering simulation, confirming the reliability and engineering applicability of the established model.

## 1. Introduction

In the manufacturing process of GH4169 alloy turbine disks, the quenching treatment after solution treatment is a critical step that determines the final mechanical properties and microstructural state of the component [1]. Its primary purpose is to suppress the precipitation of harmful phases through rapid cooling, such as water quenching, thereby providing a favorable matrix condition for the subsequent precipitation of strengthening phases during aging treatment [2]. However, quenching is inevitably accompanied by significant non-uniform cooling. Owing to the large size and complex geometry of turbine disks, different regions exhibit distinct cooling rates, leading to pronounced temperature gradients within the component. The incompatibility of thermal contraction induced by these temperature gradients further generates thermal stress and plastic deformation, eventually introducing high levels of residual stress into the turbine disk [3].

Quenching residual stress persists throughout the entire life cycle of turbine disks, including manufacturing, machining, and service [4]. During subsequent machining, the redistribution and release of residual stress can cause distortion of the disk, thereby reducing machining accuracy and increasing manufacturing cost [5]. During service, the coupling of residual stress with external loading can significantly deteriorate the overall mechanical performance and shorten the service life of the turbine disk [6,7]. Therefore, accurate quantification of the formation and distribution of residual stress during quenching of GH4169 superalloy turbine disks, clarification of its evolution behavior and formation mechanism, and effective control of residual stress through process optimization are of great theoretical significance and engineering value for improving machining accuracy, extending service life, and ensuring the safe and reliable operation of aero-engines.

At present, the research methods for residual stress mainly include experimental measurement and numerical simulation. Experimental methods mainly involve the hole-drilling method, contour method, X-ray diffraction, and neutron diffraction [8,9,10,11]. Liu et al. [12] measured the quenching residual stress in IN718 disks quenched with different media, including water, oil, air and insulation, using neutron diffraction. Their results showed that compressive stress was generated in the surface regions of the water-quenched, oil-quenched, and air-cooled samples, while balancing tensile stress existed in the interior. In contrast, no obvious residual stress was observed in the insulation-cooled sample. Although these experimental methods can directly provide residual stress data, they generally suffer from high cost, low efficiency, and limited applicability. In particular, they are difficult to use for full-field characterization of the three-dimensional residual stress distribution inside turbine disks and are even less capable of capturing the evolution of residual stress during quenching.

With the development of high-performance computing and finite element theory, numerical simulation has become an important tool for investigating the formation mechanism and evolution behavior of residual stress because of its high efficiency, low cost, good repeatability, and capability of visualizing the full-field stress distribution. It can effectively compensate for the limitations of experimental methods. Accordingly, the current research strategy has gradually shifted toward a mode dominated by numerical simulation and assisted by experimental validation. Dye et al. [13] simulated the quenching residual stress distribution in IN718 bars under water, oil, and air cooling conditions and validated the results by neutron diffraction, showing good agreement between simulation and experiment. Rist et al. [14] measured the internal residual stress along five characteristic paths in a water-quenched IN718 turbine disk using neutron diffraction and established a thermo-mechanically coupled finite element model considering temperature-dependent material properties. The simulated results were generally consistent with the experimental trends. Gong et al. [15] investigated the quenching residual stress in 7050 aluminum alloy using finite element simulation in combination with the contour method, X-ray diffraction, and layer removal method. Fan et al. [16] predicted the quenching residual stress in a nickel-based superalloy specimen by finite element simulation, and the results were in good agreement with those obtained by the contour method. Fatmi et al. [17] established a three-dimensional finite element model and a residual stress reconstruction model based on eigenstrain theory to study the residual stress field around cold-expanded holes in IN718 and compared the results with neutron diffraction measurements. These studies demonstrate that the combination of finite element simulation and experimental measurement can not only improve the accuracy of residual stress characterization, but also reduce research cost and shorten the development cycle, thus providing a reliable basis for process optimization.

Although some studies have been conducted on the finite element simulation of quenching residual stress in GH4169 superalloy components, several limitations still remain. On the one hand, some existing models did not fully consider the temperature dependence of thermophysical material properties or the influence of simplified heat-transfer boundary conditions, resulting in considerable deviations between simulated and experimental results [18]. On the other hand, systematic investigations on the distribution characteristics, formation mechanism, and evolution behavior of quenching residual stress in turbine disks are still insufficient, making it difficult to provide precise guidance for practical process optimization.

In addition, traditional phenomenological constitutive models (e.g., Johnson–Cook) often struggle to accurately reproduce the complex temperature and strain rate dependent flow behavior of GH4169 over wide thermomechanical ranges. Few studies have integrated data driven neural network constitutive descriptions into turbine disk quenching finite-element frameworks, and the corresponding model implementation, verification and physical rationality still need further exploration.

Therefore, in this work, finite element simulation was carried out to investigate the residual stress generated during quenching of GH4169 superalloy turbine disks. A finite element model of the quenching process was established in ABAQUS2021 to systematically analyze the formation mechanism, evolution behavior, and distribution characteristics of quenching residual stress. The simulation results were further compared with experimental data to validate the accuracy and reliability of the model. The present study is expected to provide theoretical support and technical guidance for quenching process optimization, residual stress control, and performance improvement of turbine disks.

## 2. Materials and Finite Element Modeling Procedures

### 2.1. Materials

GH4169 superalloy was used in this study. The main chemical composition of the alloy was 20 wt.% Cr, 3 wt.% Mo, 5 wt.% Nb, 0.5 wt.% Al, and 1 wt.% Ti, with Ni as the balance. The turbine disk forging was fabricated through a triple-melting route, followed by homogenization, cogging, fast forging, and die forging. The forged disk was then solution treated at 980 °C for 1 h and subsequently water-quenched to room temperature.

### 2.2. Determination of Material Parameters

Material parameters of GH4169 superalloy were obtained by combining experimental measurements and theoretical calculations. Cubic specimens (20 × 20 × 20 mm^3^) were ultrasonically cleaned in anhydrous ethanol, dried in a vacuum oven at 60 °C for 2 h, and cooled to room temperature. The room-temperature density was measured by the Archimedes method using an electronic balance, with three repeated measurements averaged for each specimen. The density at elevated temperatures was calculated from the measured thermal expansion coefficient. The thermal expansion coefficient was measured using a NETZSCH DIL 402SE (NETZSCH, Selb, Germany) horizontal push-rod dilatometer. Cylindrical specimens (Φ6 × 25 mm) were tested under Ar from room temperature to 1000 °C at a heating rate of 5 °C/min. Each specimen was measured three times, and the average value was used. The specific heat capacity was measured using a NETZSCH DSC 300 (NETZSCH, Selb, Germany) differential scanning calorimeter. Disk specimens (Φ4 × 0.5 mm) were tested in alumina crucibles under Ar from room temperature to 1000 °C at 10 °C/min, using high-purity indium for calibration. Each specimen was tested twice, and the average value was taken, with the experimental error controlled within ±2%. The thermal conductivity was determined using an LFA 467 (NETZSCH, Selb, Germany) laser flash apparatus. Polished disk specimens (Φ12.7 × 2.5 mm) were tested under vacuum from room temperature to 1000 °C, and each temperature point was measured three times. The thermal conductivity was calculated from the thermal diffusivity, specific heat capacity, and density.

**Figure 1:**
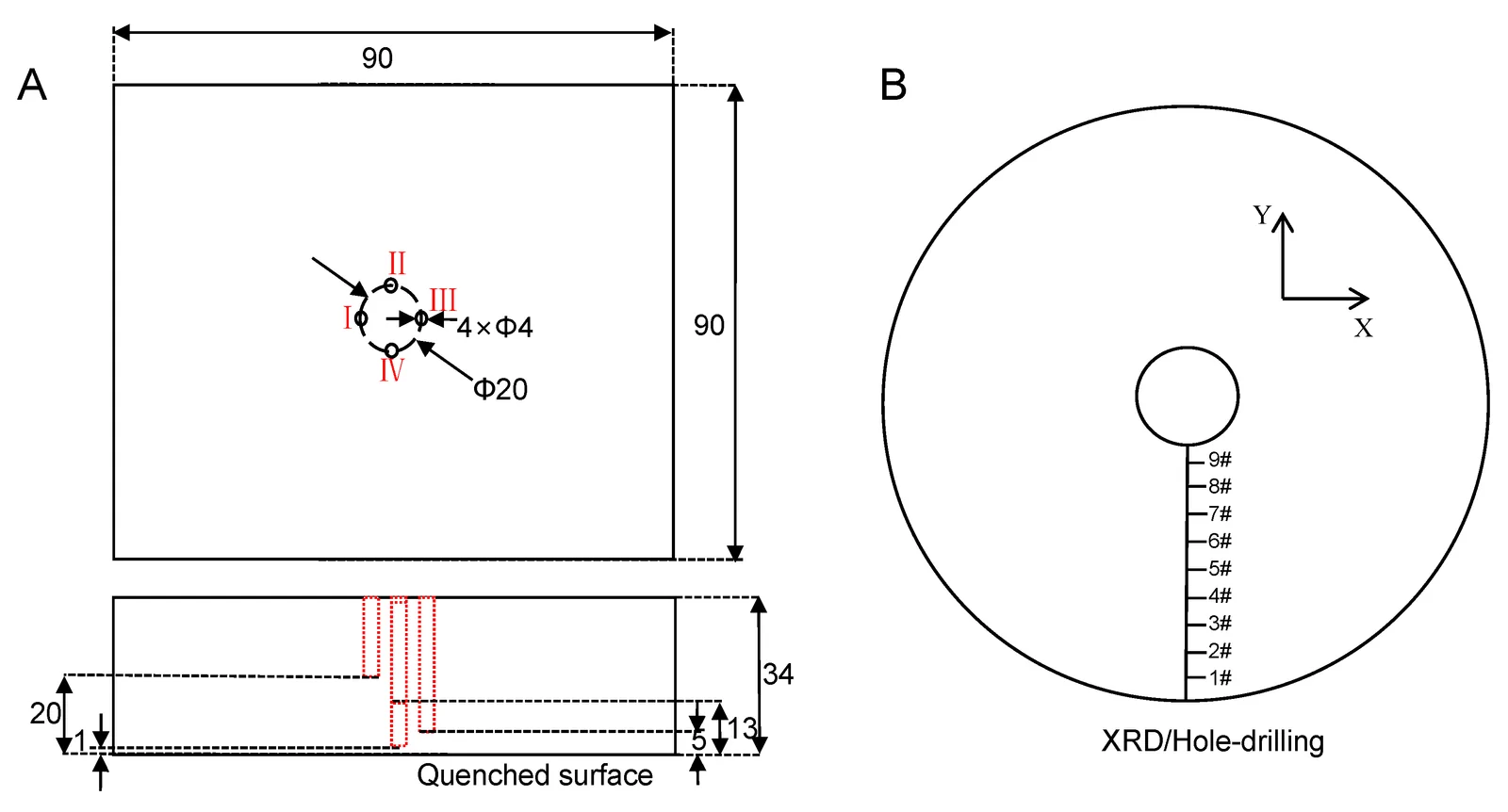
One-end quenching specimen and residual stress measurement locations. (**A**) Schematic of the one-end quenching specimen (unit: mm). (**B**) Locations of residual stress measurement points.

The heat transfer coefficient between GH4169 alloy and water was determined by a one-end quenching experiment. As shown in Figure 1A, four blind holes with different depths (I-14 mm, II-21 mm, III-29 mm, IV-33 mm) were machined near the center of the non-quenched surface along the normal direction, and thermocouples were embedded to record the temperature evolution during quenching. After holding at 980 °C for 1 h, the specimen was quenched from one end, and the temperature responses at different depths were collected. The heat transfer coefficient was identified using an inverse heat transfer method based on the measured temperature data. A quadratic fitting function was established with depth as the independent variable to reconstruct the transient temperature field within the specimen. The error between the simulated and experimental temperature curves was then used to iteratively correct the heat transfer coefficient, and the final heat transfer coefficient between GH4169 alloy and water was obtained [19].

The stress–strain curves of GH4169 alloy were obtained at temperatures ranging from 20 to 1000 °C and strain rates of 0.01, 0.001, and 0.0001 s^−1^. After preprocessing, including normalization and dataset partitioning, the data were used to train a BP neural network model, in which temperature, strain rate, and strain were taken as inputs and stress was taken as the output. The BP neural network adopted a 3-15-7-1 multi-layer feedforward architecture: 3 input neurons (temperature, strain rate, equivalent plastic strain), two hidden layers with 15 and 7 neurons respectively, and one output neuron for flow stress. The Swish activation function was used for hidden layers. The total dataset contained 21,000 groups of stress–strain data, randomly split into 75% training set, 25% validation set. AdamW optimizer was utilized for weight bias iteration, with an initial learning rate of 0.0005 and maximum epoch of 6000; training was terminated when the validation set loss ceased to decrease for 200 consecutive epochs. Root mean square error (RMSE) and mean absolute percentage error (MAPE) were selected as evaluation metrics to further quantitatively analyze the agreement between the predicted and experimental values, and assess the prediction accuracy and fitting performance of the model. After defining the hyperparameters, the model was trained through forward propagation and back propagation. The prediction error was evaluated by the loss function, and the network weights and biases were iteratively updated using an optimizer until the preset criterion was satisfied. A neural network-based constitutive model of GH4169 alloy was thus established.

The trained BP neural network constitutive model was implemented into ABAQUS via the UMAT user material subroutine under the von Mises yield criterion. At each material integration point, temperature, strain rate and accumulated equivalent plastic strain were passed into the neural network inference module to output the current flow stress, which was returned to the finite element solver for plastic flow calculation.

### 2.3. Residual Stress Measurement

X-ray diffraction and the hole-drilling method were used to characterize the surface and near-surface residual stresses of the turbine disk forging, respectively. The heat treatment process and geometric dimensions of the disk were consistent with those of the finite element model. Based on the structural symmetry and stress distribution characteristics, nine measurement points were arranged along the radial direction on the upper surface of the disk, with a spacing of 10 mm, as shown in Figure 1B. Point 1# and point 9# were located 10 and 4 mm from the edge, respectively. Residual stresses were measured in the hoop (X) and radial (Y) directions. The surface residual stresses at all nine points were measured by X-ray diffraction, while the near-surface residual stresses at eight points excluding point 9# were measured by the hole-drilling method, since the proximity of point 9# to the edge could reduce the measurement accuracy.

### 2.4. Finite Element Model

A finite element model of the quenching process for the GH4169 alloy disk forging was established in ABAQUS. Since the nucleation and growth of strengthening precipitates in GH4169 alloy generally require several hours of holding within the aging temperature range, whereas quenching is a rapid cooling process with a short duration and a high cooling rate, the cooling rate of water quenching in this study is higher than the critical cooling rate for γ″/γ′ precipitation of GH4169, so the precipitation induced strength evolution and volume shrinkage can be reasonably neglected during quenching. The δ phase is a stable or metastable phase whose dissolution requires sufficient thermal exposure. In the present process, the solution treatment is performed at 980 °C for 1 h, which is below the δ solvus and the holding time is relatively short; therefore, complete δ dissolution is not expected during the solution treatment. And the subsequent water quenching is a rapid cooling process with a very short duration. Therefore, δ dissolution during quenching is even less significant than during solution treatment. For this reason, δ dissolution is not expected to contribute significantly to the strain evolution during quenching, and it is not included in the present constitutive model. Creep deformation at 980 °C during the very early quenching stage was neglected in this model. First, the total duration for which the disk remains at temperatures above 900 °C during water quenching is very short, the high-temperature window relevant for creep is limited to the initial stage of quenching. Second, the yield strength of GH4169 at 980 °C is low, the driving stress for creep is therefore limited and decreases quickly as the temperature falls. Third, taking a conservative upper-bound creep rate for GH4169 at 980 °C under a stress level of 100 MPa and assuming that the high-temperature exposure lasts for a few seconds, the accumulated creep strain is estimated to be 10^−5^–10^−4^. This is lower than the thermal-contraction-induced plastic strain accumulated in the surface and core regions, whose maximum equivalent plastic strain reaches 6.018% in the present simulation. Therefore, the creep strain can be reasonably neglected in the present water-quenching analysis. This work adopts sequentially coupled thermo-mechanical analysis. The heat generated by plastic deformation is less than 1% of the total heat flux during rapid water quenching. Therefore, the mechanical-to-thermal feedback term can be omitted, justifying the one-way sequential coupling scheme. Fully coupled thermo-mechanical trial simulations on a representative local sector were additionally performed; the differences in temperature field and residual stress amplitude between sequential coupled and fully coupled results were less than 3.2%, and the computation time of fully coupled thermo-mechanical analysis is much longer than that of sequentially coupled analysis, confirming the rationality of sequentially coupled strategy.

Based on this assumption, a sequentially coupled thermo-mechanical method was employed. The coupling between the temperature field and the stress field was realized through a predefined field. First, a transient heat transfer analysis was performed to obtain the temperature field of the disk, and then the temperature field was imported into the mechanical analysis as a predefined thermal load to calculate the evolution of the residual stress field.

**Figure 2:**
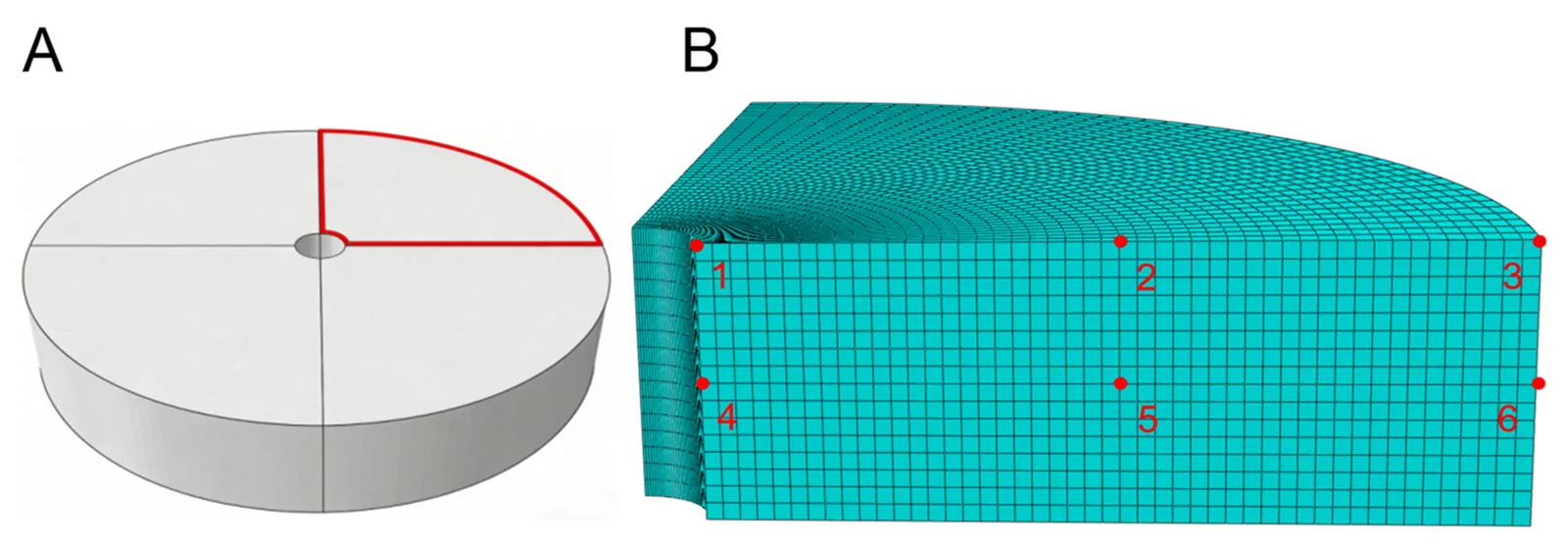
Finite element model establishment of GH4169 alloy turbine disk forging. (**A**) Position of the model in the full disk. (**B**) Mesh configuration and reference points (1−6).

The geometric dimensions of the finite element model were identical to those of the disk used in the residual stress measurement experiments, with a thickness of 33 mm, an outer diameter of 206 mm, and a central hole diameter of 18 mm. This ensured direct comparison between the simulation and experimental results and provided a consistent dimensional basis for model validation. Considering the structural symmetry of the disk, one quarter of the full disk was modeled to reduce the computational cost, as shown in Figure 2A. Symmetry boundary conditions were imposed on the two symmetry cutting planes (normal direction displacement constrained, tangential directions free). No additional mechanical clamping constraints were applied on the disk; only rigid body motion was suppressed by a single reference point constraint to avoid over constraint.

The same set of temperature-dependent convective heat transfer coefficients obtained from inverse quenching experiments was applied to the outer cylindrical surfaces, upper/lower flat surfaces and inner hole surfaces, neglecting boundary condition differences caused by coolant flow variations on each surface. The temperature-dependent heat transfer coefficient h characterizes the film boiling and nucleate boiling regimes. This simplification enables qualitative analysis of the overall temperature evolution of the disk; the simulation only captures the general cooling trend and yields certain errors in local surface temperature gradients, which leads to inevitable deviations in the final calculation results.

The meshed model is shown in Figure 2B. For the temperature field simulation, three-dimensional eight-node linear heat transfer brick elements (DC3D8) were used, while for the stress field simulation, three-dimensional eight-node linear fully integrated brick elements (C3D8) were adopted. Since C3D8 elements use full integration, they do not suffer from hourglass modes. The relevant numerical concerns for this element type are volumetric locking and shear locking under bending-dominated or nearly incompressible conditions. In the present quenching analysis, the deformation is dominated by thermal contraction and the associated plastic flow, the stress state is predominantly compressive/tensile rather than bending-dominated, and the accumulated equivalent plastic strain is moderate (maximum about 6.018%). Element quality checks were performed, and the aspect ratios were controlled within 1–3. A mesh convergence study with four mesh schemes (14,226, 22,742, 32,336 and 45,118 elements) showed that when the element number exceeded 32,336, the maximum hoop residual stress deviation was below 2.1%. Therefore, the mesh with 32,336 C3D8 elements was selected for the formal simulation. The fully integrated C3D8 element was chosen because it provides a stable and accurate description of the temperature gradient and plastic strain evolution during quenching, and no significant locking-induced over-stiffening was observed in the present low-distortion quenching problem. The six reference points marked in Figure 2B were used for subsequent analysis. The initial temperature was set to 980 °C, corresponding to the solution treatment temperature of GH4169 alloy, and the temperature of the quenching medium was set to 20 °C. The convective boundary condition between the specimen surface and the quenching medium was defined according to the relationship between the heat transfer coefficient and temperature, so as to accurately simulate the heat transfer process during quenching. Based on prior literature and our repeated heat treatment experiments, the yield strength of GH4169 drops significantly when heated above 900 °C. At 980 °Cand a tensile strain rate of 0.01 s^−1^, its yield strength is less than 200 MPa, accompanied by rapid stress relaxation. Therefore, the residual stress accumulated in previous stages can be regarded as fully released during the 1 h solution treatment at 980 °C, and the forging undergoes sufficient high-temperature stress relaxation; hence the initial stress field is set to zero.

## 3. Experimental Results

### 3.1. Material Parameters of GH4169 Alloy

**Figure 3:**
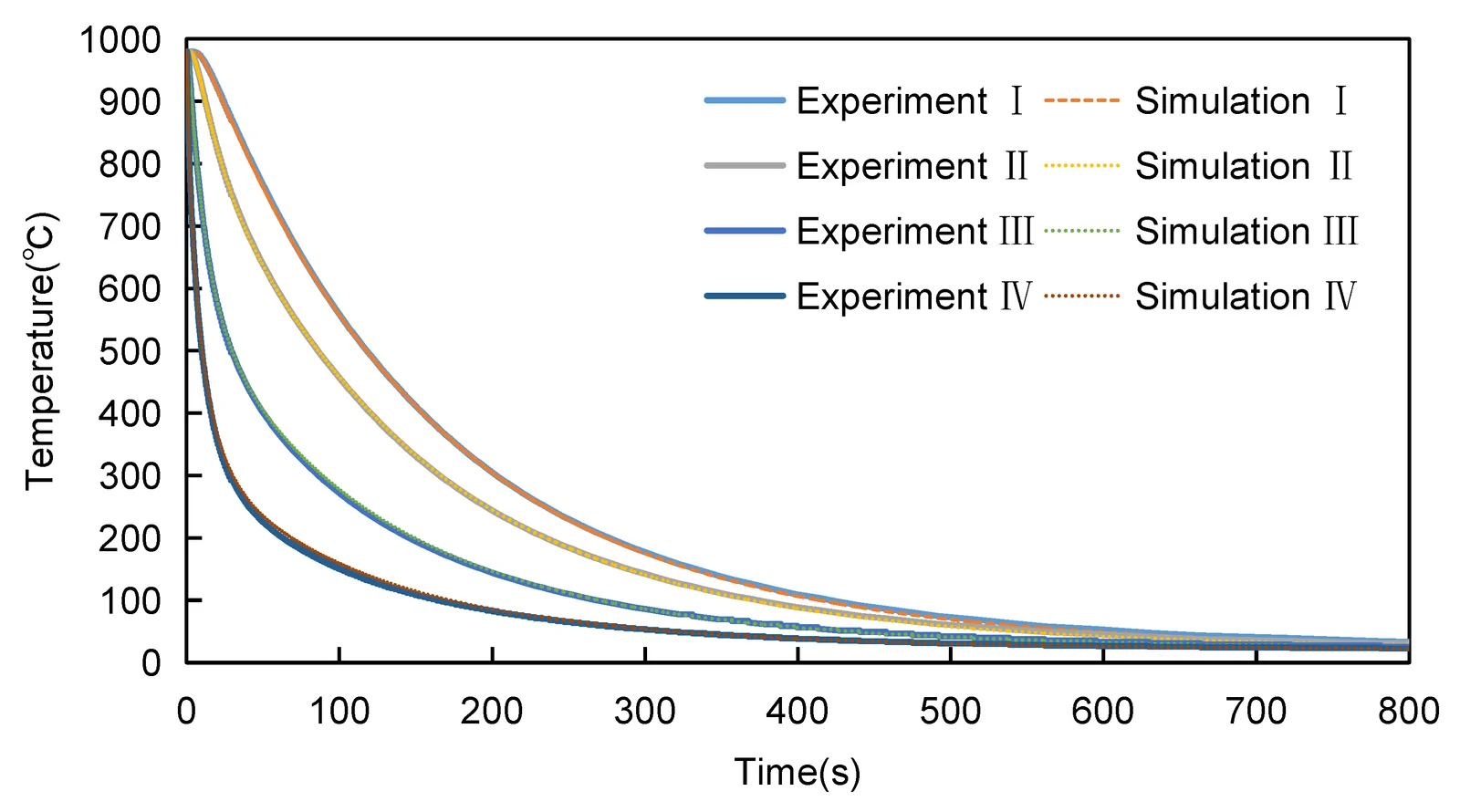
Comparison of simulated and experimental cooling curves at different depths.

The material parameters of GH4169 alloy, including density, thermal conductivity, specific heat capacity, thermal expansion coefficient, and heat transfer coefficient, are listed in Table 1. Based on these parameters, a finite element model of one-end quenching was established in ABAQUS, and the simulated cooling curves at different depths (see in Figure 1A) were obtained. A comparison between the simulated and experimental cooling curves is shown in Figure 3. It can be seen that the simulated curves agree well with the experimental ones at all measured depths, indicating good consistency between the simulation and experiment. The mean absolute percentage error (MAPE) at different depths was further calculated to evaluate the simulation accuracy, and the values were 0.64%, 0.60%, 0.99%, and 2.48%, respectively. All MAPE values were below 3%, which is within the acceptable range for engineering numerical simulation. These results demonstrate that the heat transfer coefficient determined from the cooling curves at different depths in the one-end quenching experiment using the inverse heat transfer method is reliable and can be used for subsequent simulation of the quenching temperature and stress fields.

### 3.2. Stress–Strain Curves Predicted by the BP Neural Network Constitutive Model

**Figure 4:**
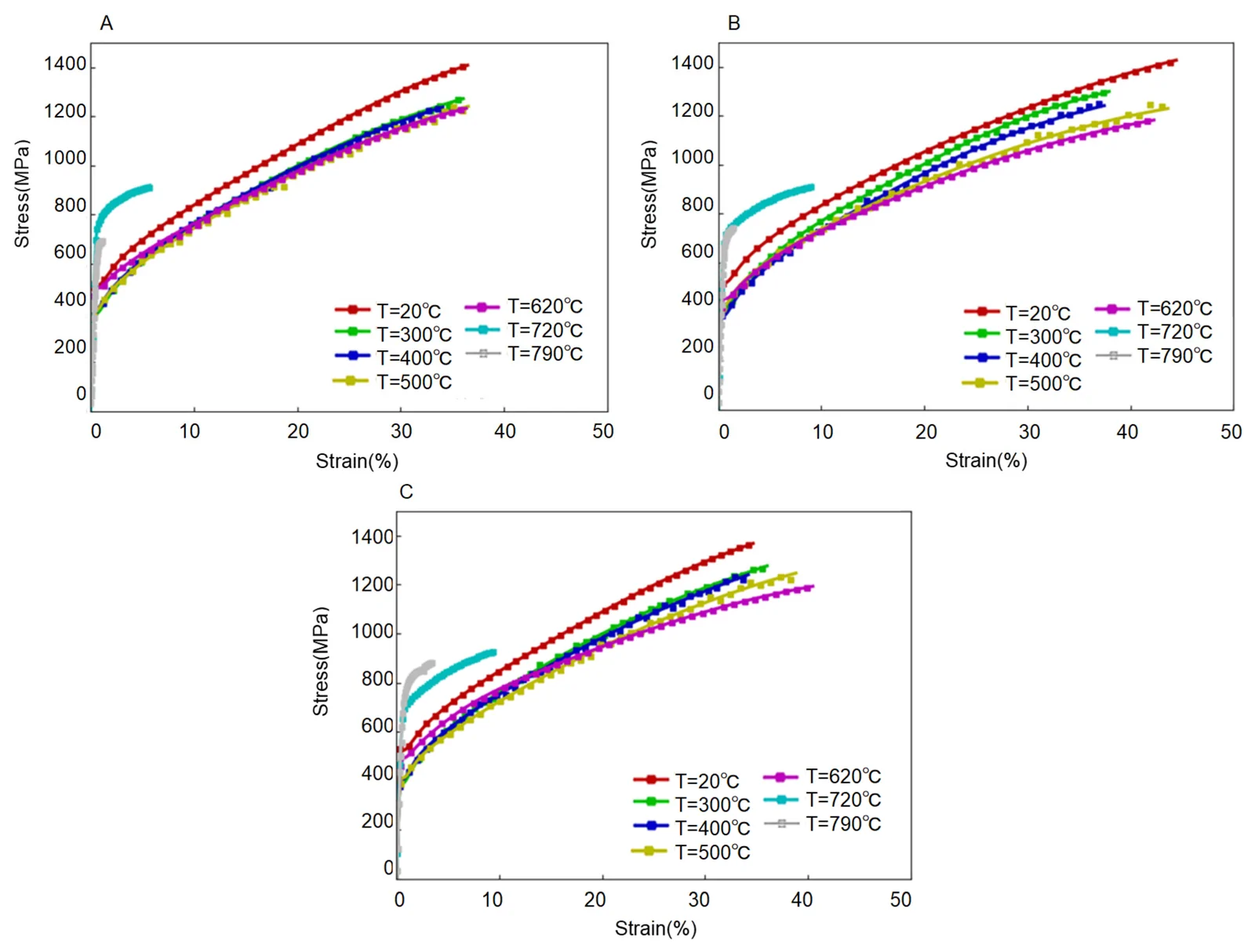
Comparison between the stress–strain curves predicted by the quenching constitutive model (lines) and the experimental data (points): (**A**) 0.0001 s^−1^; (**B**) 0.001 s^−1^; (**C**) 0.01 s^−1^.

After completion of the BP neural network constitutive model training, the predicted stress–strain curves were obtained and compared with the experimentally measured stress–strain curves, as shown in Figure 4. It can be seen that the predicted curves are in good agreement with the experimental data points, with highly consistent variation trends and good overall fitting quality. In all characteristic deformation stages, the predicted values match the experimental values well, without obvious systematic deviation. To further quantitatively evaluate the agreement between the predicted and experimental results, the root mean square error (RMSE) and mean absolute percentage error (MAPE) of the constitutive model were calculated to be 7.270 MPa and 1.19%, respectively. Both error metrics are at low levels, indicating that the established BP neural network constitutive model can accurately describe the stress–strain response of GH4169 superalloy, with high fitting accuracy and good prediction reliability.

### 3.3. Simulation Results of the Temperature Field During Quenching

**Figure 5:**
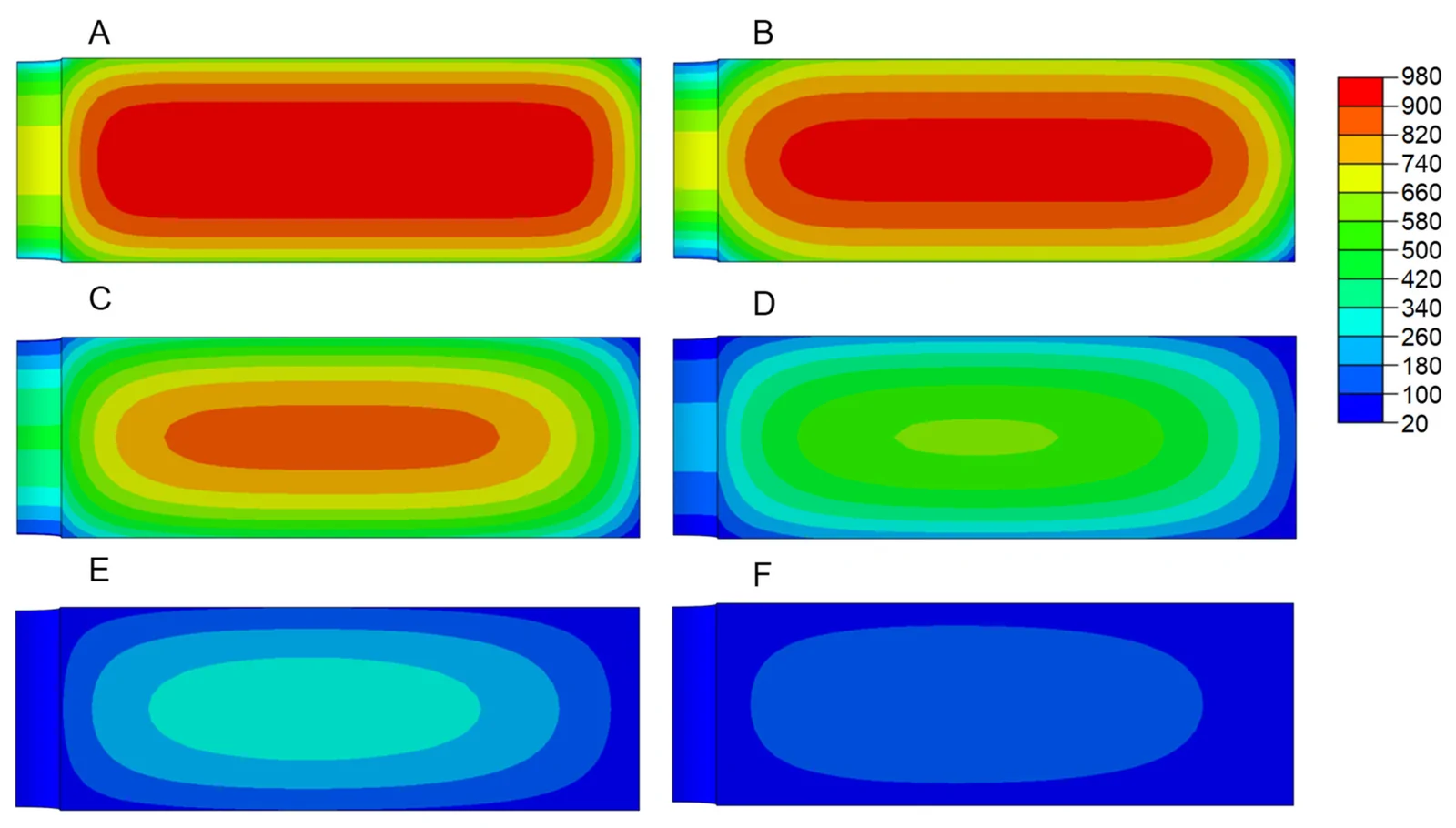
Temperature distribution of the disk at different times during quenching process (unit: °C): (**A**) 5 s; (**B**) 10 s; (**C**) 20 s; (**D**) 40 s; (**E**) 70 s; (**F**) 100 s.

To clarify the temperature evolution of the disk during quenching, the temperature contours at different times and the temperature histories of the six reference points (see in Figure 2B) are shown in Figure 5. During quenching, the temperature drop rate is the highest on the outer and bore surfaces of the disk, especially in the corner regions. In contrast, the cooling process within the disk proceeds relatively slowly. This occurs because the disk surfaces are in direct contact with the quenching medium, leading to intense convective heat transfer. Given the large diameter and thickness of the disk, internal heat must be gradually transferred to the surface via thermal conduction before dissipation; the longer heat transfer path results in greater time consumption, and consequently the temperature variation in the interior significantly lags behind that at the surface.

Spatial differences in cooling rate at various locations create a substantial temperature gradient between the inner and outer portions of the disk. Approximately 20 s after quenching initiation, the maximum temperature difference between the surface layer and the interior reaches 760 °C. Such an extreme temperature difference acts as a key driving force for the generation and evolution of quenching residual stress. At high temperatures, the material possesses low yield strength, and non-uniform thermal shrinkage deformation can be partially relieved through plastic flow. As the temperature continues to decrease, the material yield strength rises markedly. Thermal deformation generated in the subsequent cooling stage is rigidly constrained by regions that have undergone prior plastic deformation, and free coordinated deformation is prohibited. When the model is fully cooled to room temperature and the external temperature-induced loading disappears, the internal stresses trapped by deformation constraints within the disk form a self-equilibrated state. This stress field is defined as the quenching residual stress field, whose distribution and magnitude are directly governed by the accumulation and constraint effect of non-uniform elastoplastic deformation developed during cooling.

### 3.4. Simulation Results of the Residual Stress Field After Quenching

**Figure 6:**
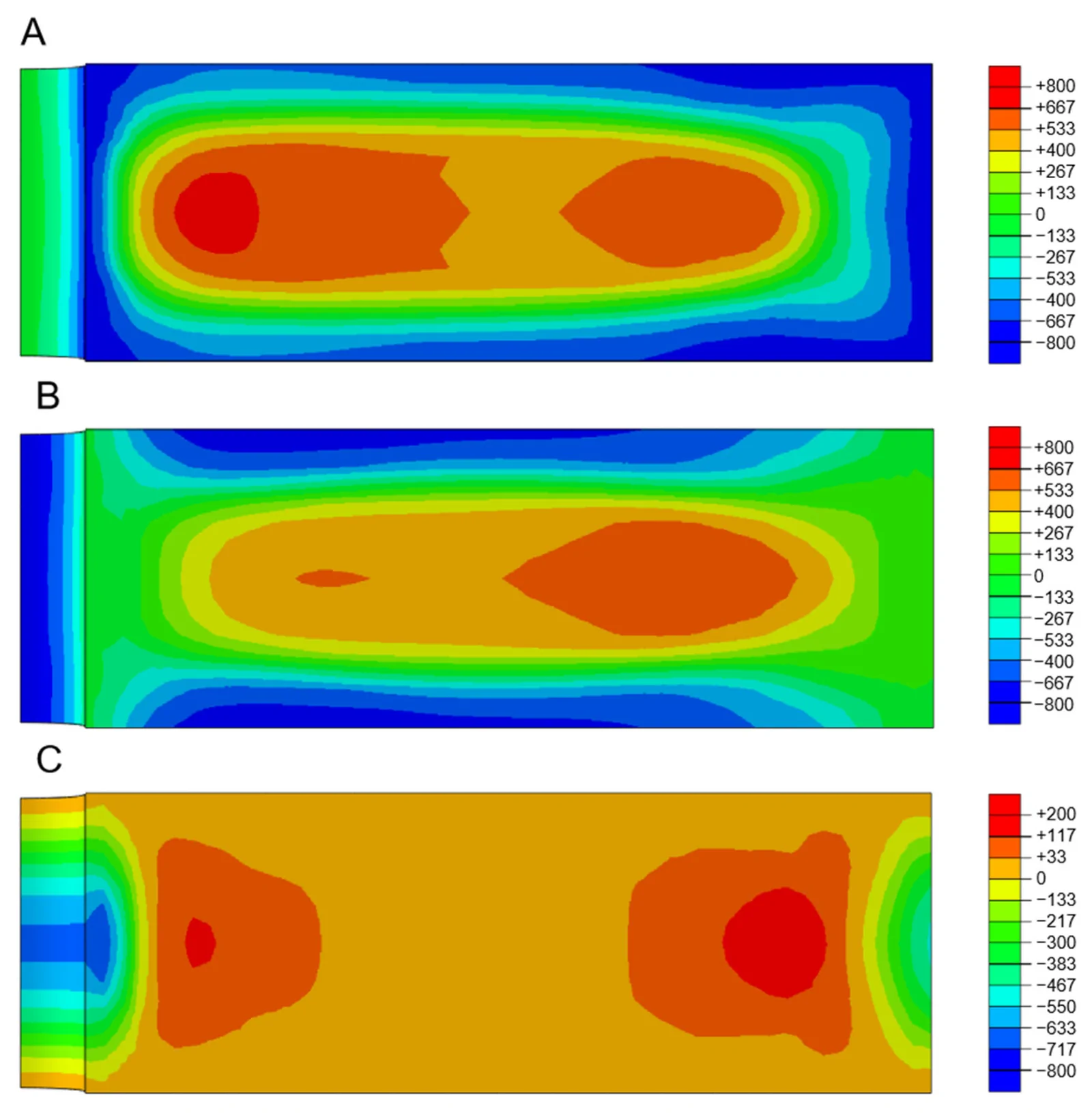
Simulated residual stress distribution in the GH4169 alloy disk after quenching (MPa). (**A**) Hoop direction. (**B**) Radial direction. (**C**) Axial direction.

Figure 6 shows the circumferential, radial, and axial residual stress distributions on the cross-section of the GH4169 alloy disk after quenching. The simulation results indicate that the circumferential residual stress is tensile in the interior of the disk and compressive in the surface region. The compressive stress gradually changes to tensile stress from the surface to the interior, showing a relatively smooth stress gradient. The radial residual stress exhibits a distribution pattern similar to that of the circumferential component, with tensile stress in the interior and compressive stress near the upper and lower surfaces, although the magnitude is significantly reduced in the regions close to the side surfaces. In contrast, the axial residual stress is generally low in magnitude and is dominated by low-level tensile stress, while compressive stress appears mainly in the regions near the side surfaces. Overall, the axial residual stress is markedly lower than the circumferential and radial components. This non-uniform residual stress distribution is closely related to the temperature gradient between the surface and interior during quenching. Rapid cooling and shrinkage of the surface region lead to the formation of a compressive stress layer, whereas the delayed cooling of the interior results in tensile stress. Together, these features constitute the typical residual stress distribution pattern induced by quenching.

**Figure 7:**
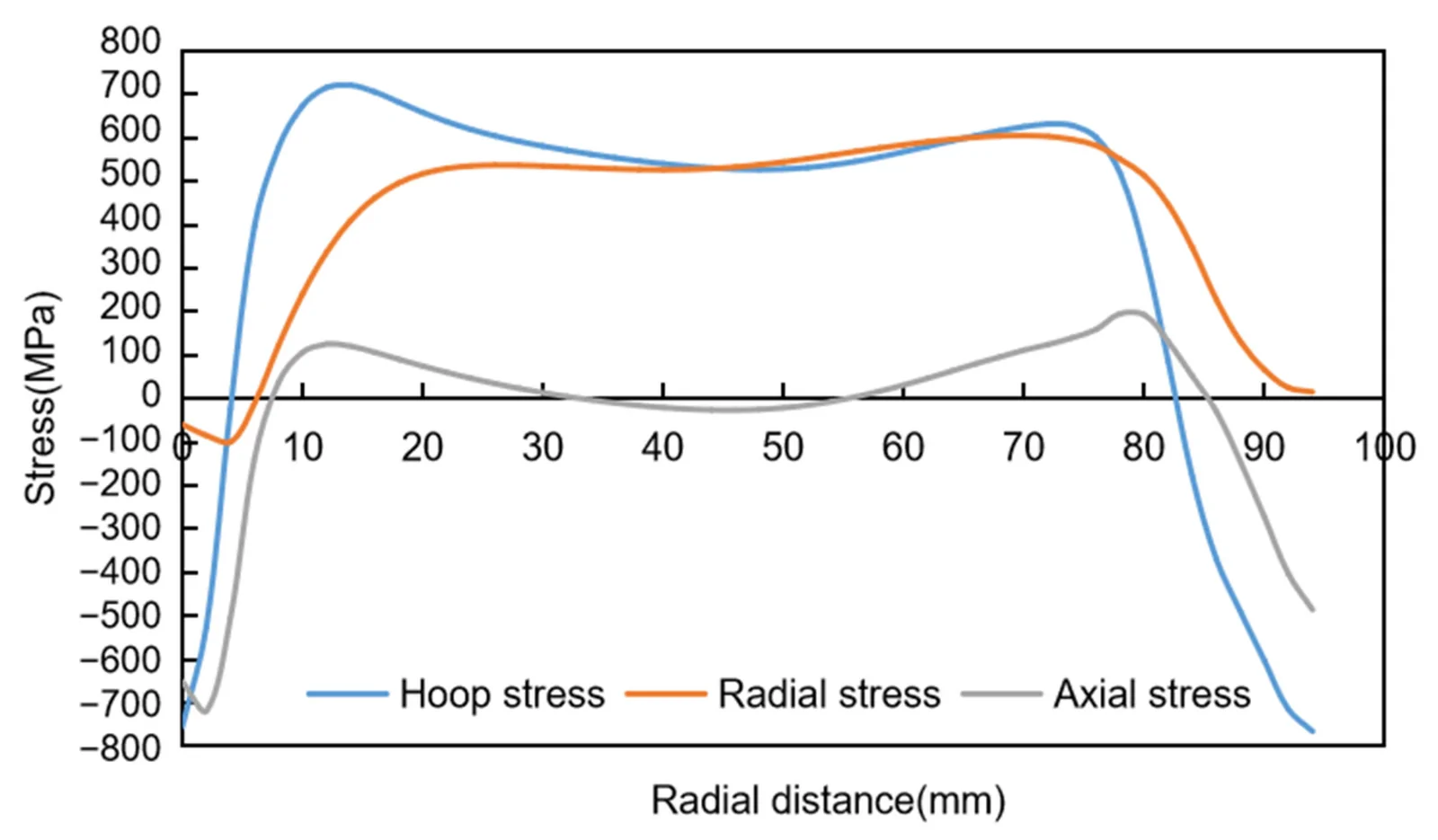
Residual stress distributions of hoop, radial, and axial directions along the radial centerline of the GH4169 disk after quenching.

Figure 7 shows the circumferential, radial, and axial residual stress distributions along the radial centerline of the disk, revealing the variation characteristics and magnitudes of different stress components along the radial direction. The circumferential residual stress exhibits a typical “inner tension–outer compression” pattern. Compressive stress appears in the regions near both side surfaces and reaches its peak at the surfaces. As the position moves from the surface toward the interior, the compressive stress gradually decreases and rapidly changes into tensile stress. In the interior region, the circumferential tensile stress increases to a high level and remains relatively stable, with local maxima appearing in the regions close to the side surfaces. The radial residual stress is dominated by tensile stress along the entire radial centerline, and its peak value is mainly located in the interior region of the disk. A slight transition to compressive stress occurs near the inner hole surface, although the compressive stress magnitude is relatively low. Near the outer surface, the radial tensile stress gradually decreases and finally approaches zero. The axial residual stress is relatively low in magnitude and is mainly tensile in the interior of the disk, with local stress peaks appearing in the regions near both side surfaces. As the position further approaches the side surfaces, the axial tensile stress decreases rapidly and transforms into compressive stress, reaching its peak value at the side surfaces. Among them, the compressive stress peak at the inner hole surface is significantly higher than that at the outer surface.

## 4. Discussion

### 4.1. Evolution of Residual Stress During Quenching

**Figure 8:**
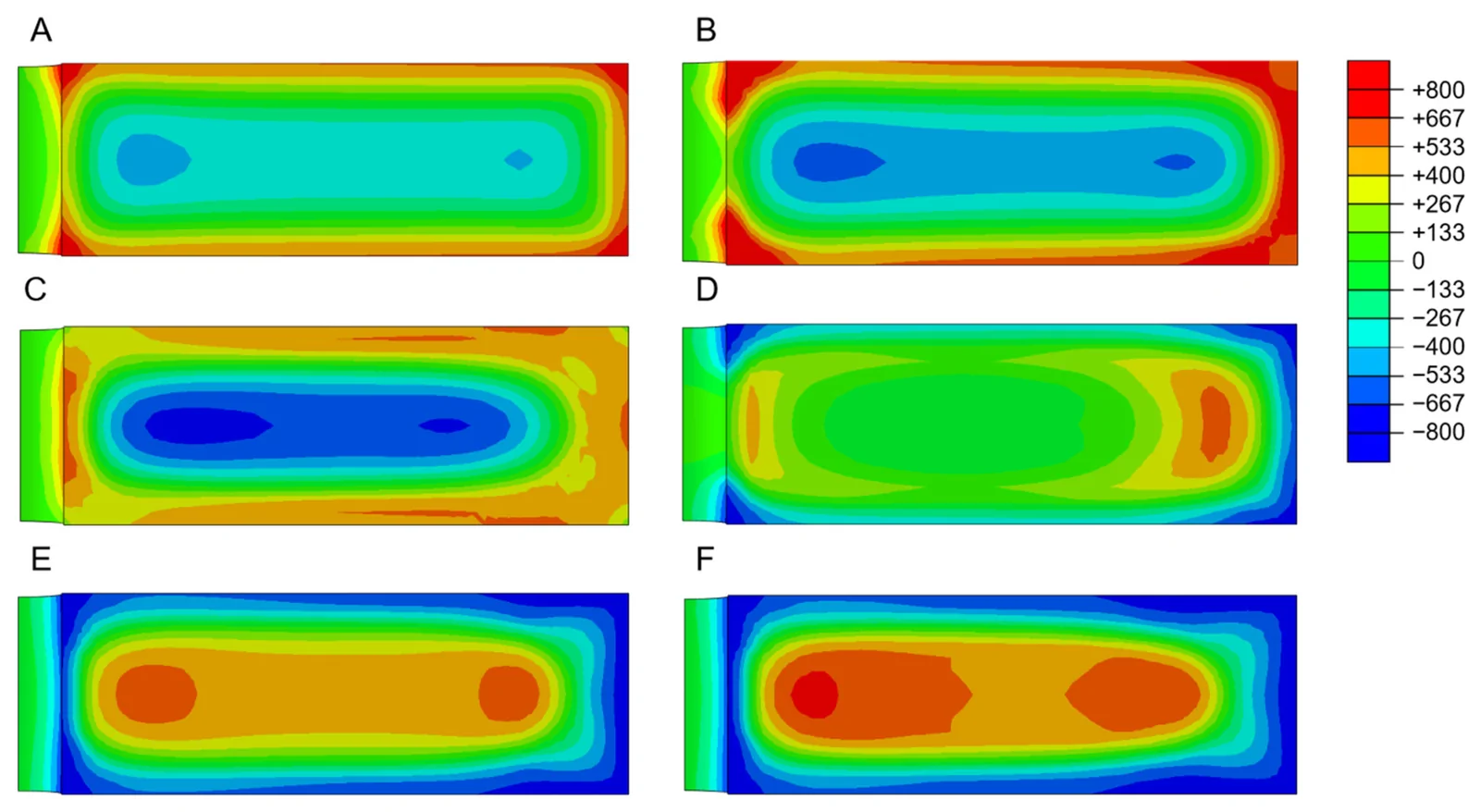
Evolution of hoop residual stress distribution in the disk at different times during quenching (unit: MPa): (**A**) 5 s; (**B**) 10 s; (**C**) 20 s; (**D**) 50 s; (**E**) 100 s; (**F**) 200 s.

**Figure 9:**
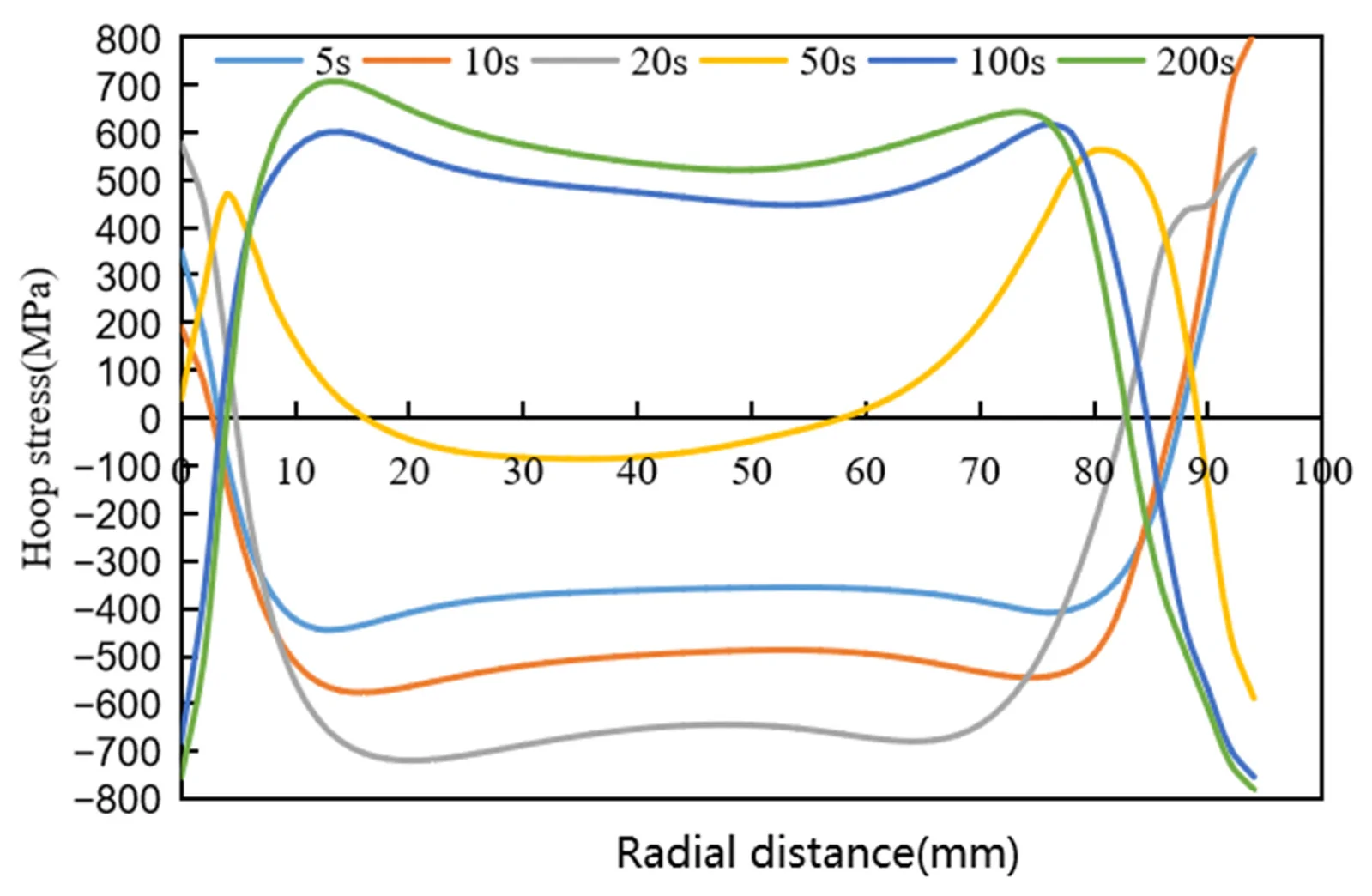
Hoop residual stress distributions along the radial centerline of the GH4169 disk at different times during quenching.

The hoop residual stress distributions on the disk cross-section and along the radial centerline at different times during quenching are shown in Figure 8 and Figure 9, respectively. At the initial stage of quenching, intense convective heat transfer occurs between the disk surface and the quenching medium, leading to rapid cooling of the surface layer, whereas the internal heat can only be transferred outward by conduction, resulting in a much slower cooling rate in the interior. Consequently, a pronounced temperature gradient is established within the disk [20]. Under this condition, the surface tends to shrink rapidly during cooling and is constrained by the relatively hot interior, thereby developing tensile stress. To maintain mechanical equilibrium, the interior is subjected to compressive stress, giving rise to an overall “inner compression–outer tension” stress distribution. As quenching proceeds, the internal temperature gradually decreases and the temperature gradient between the surface and interior is reduced. Owing to the subsequent cooling-induced shrinkage of the interior, the initial compressive stress in the interior gradually diminishes. In the later stage of quenching, with the further decrease in temperature, the yield strength of the surface layer increases and plastic deformation becomes more difficult. Meanwhile, the tensile stress generated by the continued shrinkage of the interior gradually becomes dominant. To maintain overall mechanical equilibrium, the surface stress state gradually changes from tensile to compressive, and the final residual stress distribution is characterized by an “inner tension–outer compression” pattern.

**Figure 10:**
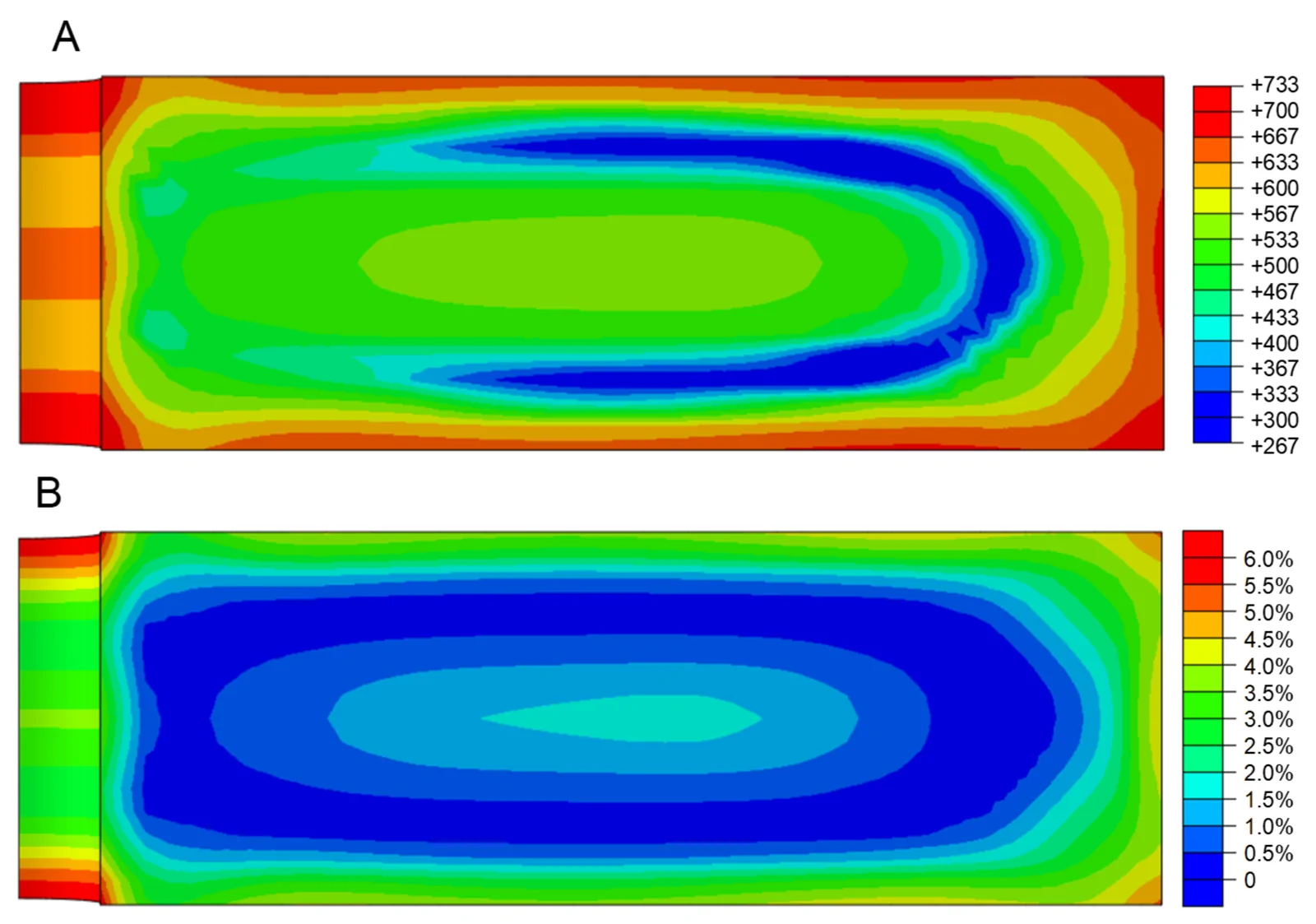
Distribution of equivalent von Mises stress and equivalent plastic strain after quenching. (**A**) Equivalent von Mises stress (MPa). (**B**) Equivalent plastic strain.

The distributions of equivalent stress and equivalent plastic strain on the disk cross-section were extracted and analyzed, as shown in Figure 10. It can be seen from the equivalent stress contour that both the surface and core regions of the disk exhibit relatively high equivalent stress levels, with the maximum value of 733.4 MPa located at the surface. Between these two high-stress regions, a distinct transition zone is observed, where the equivalent stress is significantly lower than that in the surrounding regions, forming a low-stress buffer zone that separates the high-stress regions at the surface and in the core. The distribution of equivalent plastic strain is generally consistent with that of equivalent stress. Higher equivalent plastic strain values are found in the surface and core regions, indicating more pronounced accumulation of plastic deformation in these areas. Similarly, a low equivalent plastic strain zone exists between the surface and core regions, forming a low-strain buffer zone that separates the two high-strain regions. The maximum equivalent plastic strain appears at the disk surface, with a value of 6.018%.

These distributions of equivalent stress and equivalent plastic strain are essentially the result of the combined effects of different cooling rates and mutual constraints between the surface and core during quenching [21]. The disk surface is in direct contact with the quenching medium and therefore cools most rapidly, leading to severe thermal contraction caused by the sharp temperature drop. In contrast, the core cools more slowly, and its thermal contraction lags behind that of the surface. As a result, the rapid contraction of the surface is constrained by the hotter core, which leads to significant plastic deformation and stress concentration. As quenching proceeds, the core undergoes subsequent cooling and contraction, but its free shrinkage is restricted by the already cooled surface, resulting in further plastic deformation and stress concentration. By comparison, the transition region cools at an intermediate rate between those of the surface and core, and the temperature gradient is relatively moderate. Therefore, the thermal deformation in this region is more compatible, and less plastic deformation accumulates, leading to the formation of a low-stress, low-strain buffer zone. This transition zone can effectively alleviate the stress gradient between the surface and core, serving as a buffer and separation region for both stress and strain.

**Figure 11:**
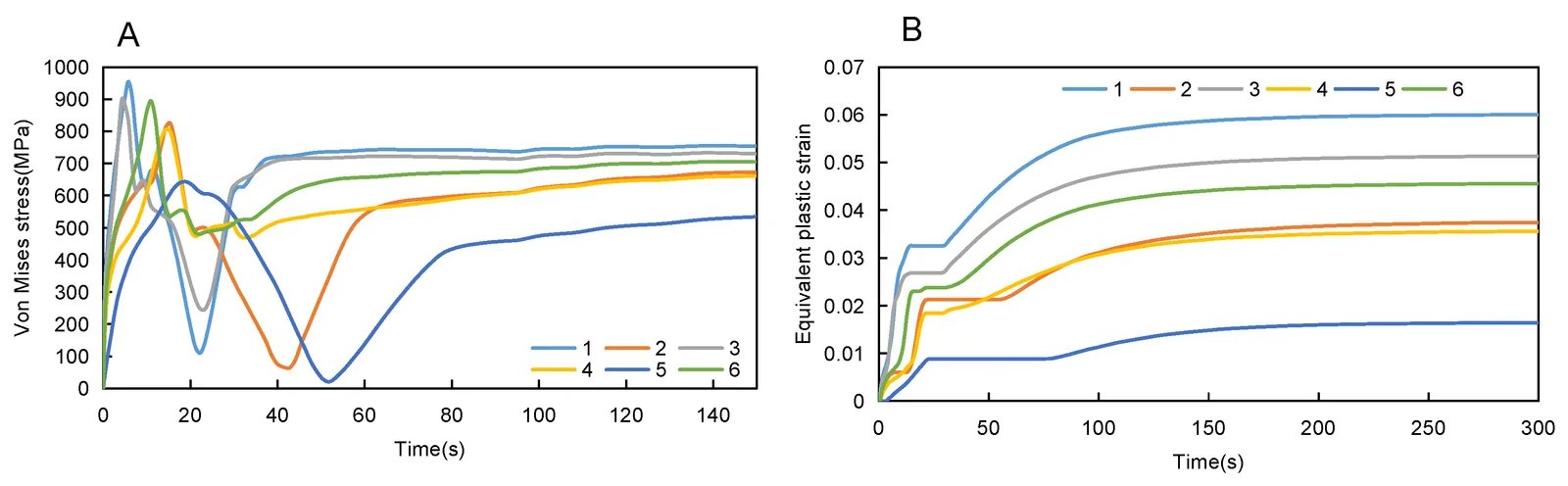
Time-dependent evolution of equivalent von Mises stress and equivalent plastic strain at reference points 1–6 during quenching. (**A**) Equivalent von Mises stress (MPa). (**B**) Equivalent plastic strain.

To more intuitively analyze the evolution of equivalent stress and equivalent plastic strain during quenching, the time-dependent curves of equivalent stress and equivalent plastic strain at six reference points (seen in Figure 2B) are presented in Figure 11. The results show that the equivalent stress and equivalent plastic strain at different locations in the disk exhibit significant spatial and temporal differences during quenching. In terms of equivalent stress evolution, all six reference points exhibit a three-stage pattern characterized by a rapid increase to a peak, a sharp decrease to a minimum, and a subsequent rise followed by stabilization. At the initial stage of quenching, the equivalent stress at each point increases rapidly to its peak with the rapid development of the temperature gradient. Then, as the stress state gradually changes from “outer tension–inner compression” to “outer compression–inner tension”, the equivalent stress decreases sharply. Afterward, it rises again and gradually stabilizes at a relatively constant level. Among the six points, points 1 and 3, located at the boundary intersections, show the most pronounced stress fluctuations. By contrast, point 5, located in the interior of the model, exhibits a relatively smoother and more delayed variation in equivalent stress, and still shows noticeable fluctuations when the stresses at the other reference points have already become stable. This is closely related to its slowest cooling rate. In terms of equivalent plastic strain evolution, all reference points show an overall trend of rapid increase, slow accumulation, and eventual saturation. A clear plateau appears in each curve, corresponding to the transition of the stress state from “outer tension–inner compression” to “outer compression–inner tension”. Meanwhile, the evolution of plastic strain shows obvious spatial differences among different positions. Owing to the faster heat dissipation and larger temperature gradient, points 1 and 3 at the boundary intersections undergo plastic deformation earlier and exhibit the largest plastic strain. In contrast, point 5 in the interior cools most slowly, and therefore shows the latest onset of plastic deformation and the lowest strain accumulation. These spatial and temporal differences in the evolution of plastic strain are highly coupled with the dynamic change in temperature gradient during quenching, and ultimately lead to the formation of residual stress in the disk.

### 4.2. Experimental Validation of Simulation Results of Residual Stress in the GH4169 Disk After Quenching

**Figure 12:**
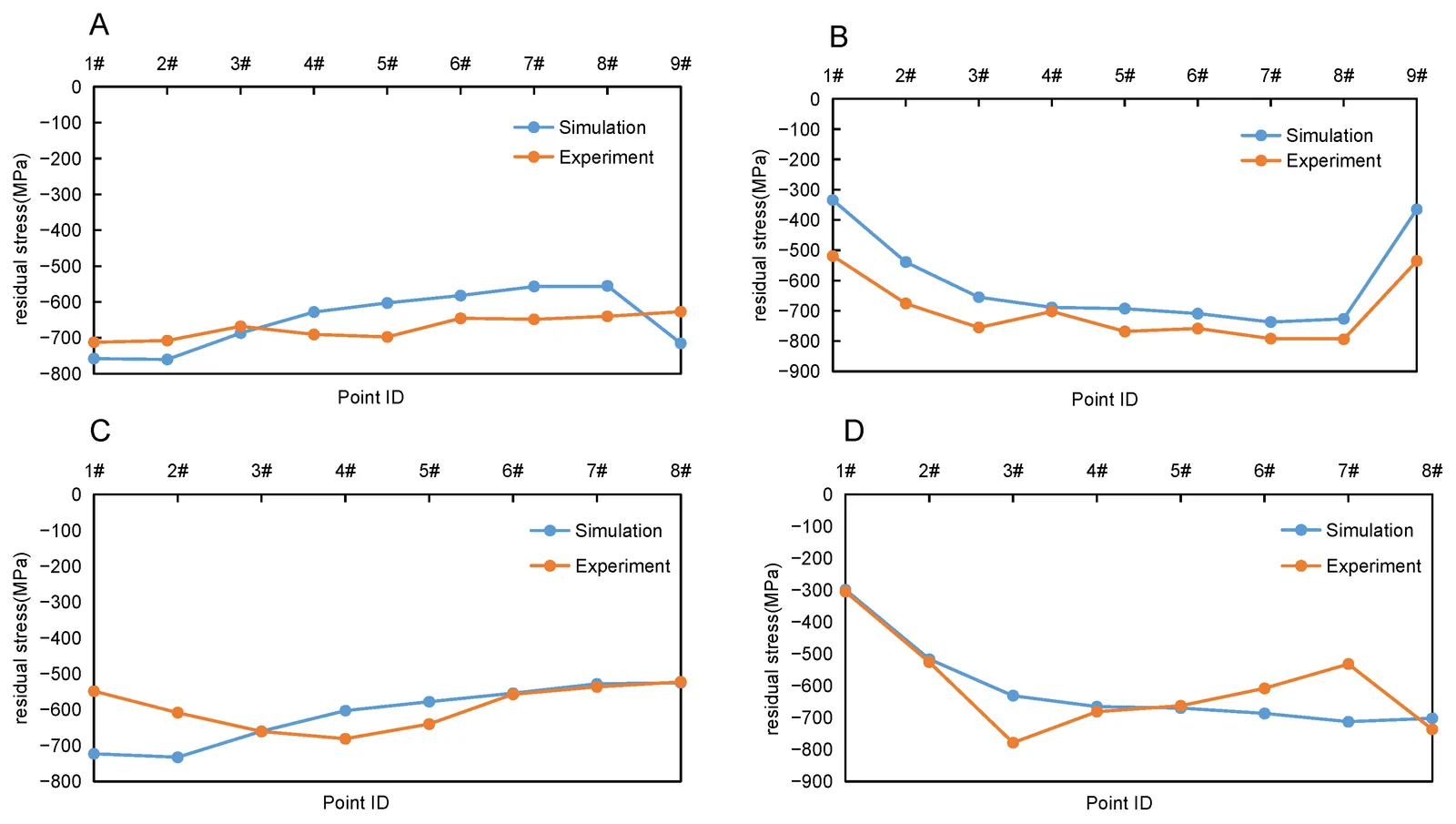
Comparison between simulated and experimental results of quenching residual stress. (**A**) X direction-XRD. (**B**) Y direction-XRD. (**C**) X direction-Hole-drilling. (**D**) Y direction-Hole-drilling.

Residual stresses on the surface and near-surface regions of the GH4169 alloy disk were measured by X-ray diffraction and the hole-drilling method, respectively, and compared with the simulated results extracted from the corresponding locations in the finite element model. The locations of the measurement points are shown in Figure 1B. The comparison data are listed in Table 2 and Table 3, and the corresponding line plots are shown in Figure 12.

To further evaluate the relative deviation between the simulated and experimentally measured residual stresses on the disk surface and near-surface, the following expression was adopted [22]:

(1) $$\bar{\delta_{j}}=\sqrt{\frac{\sum_{k=1}^{n}{\left[\sigma_{k}^{sim}-\sigma_{k}^{\exp}\right]}^{2}}{\sum_{k=1}^{n}{\left[\sigma_{k}^{\exp}\right]}^{2}}}\times 100\%$$

where j=X,Y denote the residual stress components in the X and Y directions, respectively, and $\sigma_{k}^{sim}$ and $\sigma_{k}^{exp}$ represent the simulated and experimental values, respectively.

The relative deviations of the surface residual stresses measured by X-ray diffraction in the X and Y directions are 10.59% and 15.49%, respectively, while those of the near-surface residual stresses measured by the hole-drilling method are 14.03% and 14.24%, respectively. All these deviations fall within the acceptable accuracy range for engineering numerical simulation [23,24,25]. Analysis of the above results shows that, in terms of stress state, the residual stresses on the upper surface and in the near-surface region of the quenched disk are both compressive, which is consistent with the finite element simulation results. In terms of data agreement, some deviations still exist between the measured and simulated values. For the surface residual stress, the simulated results are generally lower than the experimental values, and the main deviations are concentrated in the radial stresses at measurement points 1# and 9#, which are located near the two side surfaces. For the near-surface residual stress, nearly half of the measurement points show good agreement between the simulated and measured values, whereas relatively large deviations are observed at the remaining points. In the X direction, the larger deviations are mainly concentrated in the region near the outer surface, while in the Y direction, they are mainly concentrated in the region near the inner hole surface.

The main sources of simulation experiment deviation can be summarized as follows: (1) Simplification of actual quenching liquid flow state in finite element boundary conditions; (2) Tiny amounts of high temperature creep and minor phase transition effects that are not incorporated in the current model; (3) Measurement uncertainties originating from X-ray diffraction and hole drilling experimental procedures. Even with these factors, the overall relative deviation of ~15% satisfies engineering simulation requirements.

## 5. Conclusions

Finite element modeling was conducted to investigate the formation mechanism and evolution behavior of quenching residual stress in GH4169 alloy turbine disk forgings, and the model was validated by X-ray diffraction and hole-drilling measurements. The results show that the evolution of quenching residual stress can be divided into three stages, and the stress distribution is highly coupled with the temperature gradient and plastic deformation. During quenching, the stress state gradually changes from “inner compression–outer tension” to “inner tension–outer compression”, and the final residual stress field is characterized by compressive stress at the surface and tensile stress in the interior, with the maximum compressive and tensile stresses reaching approximately −794 and +720 MPa, respectively. Both equivalent stress and equivalent plastic strain exhibit high values in the surface and core regions and low values in the transition region, with their maximum values appearing at the disk surface, reaching 733.4 MPa and 6.018%, respectively. The spatial and temporal evolution of plastic strain is strongly dependent on the temperature gradient, indicating that temperature-gradient-induced incompatible plastic deformation is the dominant mechanism for quenching residual stress formation, while the accumulation of plastic strain directly determines the magnitude and distribution of the residual stress. Compared with conventional phenomenological constitutive models, the BP neural network-based constitutive strategy achieves higher fitting accuracy over wide temperature strain rate ranges for GH4169. The relative deviation between the simulated and measured residual stresses is about 15%, which is within the acceptable range for engineering simulation, confirming the reliability and engineering applicability of the established finite element model.

## Figures and Tables

**Table 1:** Thermophysical parameters of GH4169 superalloy.

Temperature °C	Density kg/m^3^	Thermal Conductivity W/(K·m)	Specific Heat Capacity J/(kg·K)	Thermal Expansion Coefficient 10^−6^/K	Heat Transfer Coefficient W/(K·m^2^)
20	8280	11.19	418	11.69	3852.2
100	8251	12.31	434	14.65	3956.2
200	8214	13.94	455	14.93	4029.5
300	8177	15.08	457	15.34	3846.9
400	8138	16.73	477	15.64	3513.6
500	8099	19.16	516	16.23	3236.8
600	8056	20.89	531	17.97	2812.6
700	8005	22.48	564	21.17	2628.1
800	7944	24.05	602	25.37	2463.7
900	7896	25.2	633	20.16	1751.8
1000	7849	26.96	655	20.02	989.7

**Table 2:** Comparison between simulated results and XRD measurements of quenching residual stress at different points on the disk surface.

Point	X Direction		Y Direction	
	Simulated Value MPa	Measured Value MPa	Simulated Value MPa	Measured Value MPa
1#	−757.7	−712.3	−334.0	−519.0
2#	−760.4	−707.6	−538.9	−675.9
3#	−686.7	−667.9	−654.9	−755.2
4#	−628.0	−690.6	−688.8	−702.0
5#	−602.9	−697.6	−692.6	−768.4
6#	−582.2	−645.6	−709.4	−758.5
7#	−556.9	−648.3	−737.0	−791.9
8#	−554.9	−640.0	−726.7	−794.1
9#	−715.3	−627.0	−364.7	−535.4

**Table 3:** Comparison of simulated results and hole-drilling measurements of quenching residual stress at different near-surface points of the disk.

Point	X Direction		Y Direction	
	Simulated Value MPa	Measured Value MPa	Simulated Value MPa	Measured Value MPa
1#	−723.2	−548.4	−298.9	−305.0
2#	−732.85	−608.5	−516.9	−525.8
3#	−660.5	−661.1	−631.25	−778.93
4#	−602.85	−681.23	−665.45	−681.23
5#	−577.85	−640.46	−669.85	−662.91
6#	−554.5	−557.45	−687.1	−608.45
7#	−528.1	−536.67	−713.1	−531.96
8#	−525.05	−522.57	−702.25	−736.86

## Data Availability

The original contributions presented in this study are included in the article. Further inquiries can be directed to the corresponding authors.
